# Restricted VH/VL usage and limited mutations in gluten-specific IgA of coeliac disease lesion plasma cells

**DOI:** 10.1038/ncomms5041

**Published:** 2014-06-09

**Authors:** Øyvind Steinsbø, Carole J. Henry Dunand, Min Huang, Luka Mesin, Marlene Salgado-Ferrer, Knut E. A. Lundin, Jørgen Jahnsen, Patrick C. Wilson, Ludvig M. Sollid

**Affiliations:** 1 Centre for Immune Regulation and Department of Immunology, University of Oslo and Oslo University Hospital-Rikshospitalet, 0372 Oslo, Norway; 2 Department of Medicine, Section of Rheumatology, Committee on Immunology, The Knapp Center for Lupus and Immunology Research, University of Chicago, Chicago, Illinois 60637, USA; 3 Department of Gastroenterology, Oslo University Hospital-Rikshospitalet, 0372 Oslo, Norway; 4 Division of Medicine, Department of Gastroenterology, Akershus University Hospital, 1478 Lørenskog, Norway

## Abstract

Coeliac disease (CD), an enteropathy caused by cereal gluten ingestion, is characterized by CD4^+^ T cells recognizing deamidated gluten and by antibodies reactive to gluten or the self-antigen transglutaminase 2 (TG2). TG2-specific immunoglobulin A (IgA) of plasma cells (PCs) from CD lesions have limited somatic hypermutation (SHM). Here we report that gluten-specific IgA of lesion-resident PCs share this feature. Monoclonal antibodies were expression cloned from single PCs of patients either isolated from cultures with reactivity to complex deamidated gluten antigen or by sorting with gluten peptide tetramers. Typically, the antibodies bind gluten peptides related to T-cell epitopes and many have higher reactivity to deamidated peptides. There is restricted VH and VL combination and usage among the antibodies. Limited SHM suggests that a common factor governs the mutation level in PCs producing TG2- and gluten-specific IgA. The antibodies have potential use for diagnosis of CD and for detection of gluten.

## Abstract (web summary)

Coeliac disease is characterized by an inappropriate immune response to dietary gluten proteins, involving the production of antibodies reactive to gluten. Here, the authors study the intestinal antibody response against gluten and show that gluten-specific antibodies have a low degree of somatic hypermutations.

## Graphical abstract

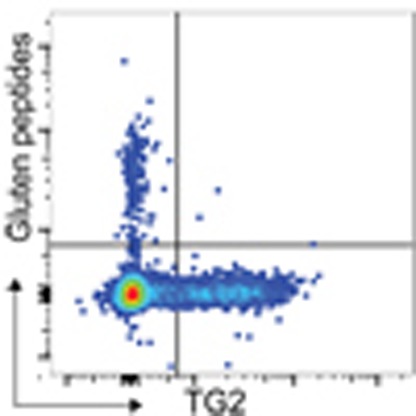

Coeliac disease (CD) is a chronic T-cell-mediated inflammatory enteropathy resulting from an inappropriate immune response to dietary gluten proteins (consisting of gliadin and glutenin components) of wheat, barley and rye. The disorder is controlled by gluten, as elimination of gluten from the diet leads to disease remission and resolution of the small intestinal disease lesion, whereas gluten provocation leads to reoccurrence of disease and pathology^1^. Distinct features of the disease are cell-mediated immunity to gluten and highly specific humoral responses to both gluten and the self-antigen transglutaminase 2 (TG2)^2^. Leukocyte infiltrations in the CD lesion reflect these immunological features; gluten-reactive CD4^+^ T cells recognizing deamidated gluten peptides in the context of the disease associated HLA-DQ2 and HLA-DQ8 molecules^3^ as well as plasma cells (PCs) secreting gluten-specific or TG2-specific immunoglobulin A (IgA) and IgM antibodies are found in the intestinal lamina propria of CD patients^4^,^5^.

Antibodies to gluten and TG2 have become increasingly important for the diagnostic workup of CD^6^,^7^. In children, histological assessment of biopsies in many cases is no longer considered mandatory due to high diagnostic accuracy of serologic assays^7^. A similar diagnostic pathway has also been suggested for adults^8^. Serologic testing is informative when subjects are consuming a gluten-containing diet. Antibodies both to gluten^9^ and TG2 (ref. 10) disappear within months after introduction of a gluten-free diet. The parallel fluctuation of antibodies to gluten and TG2 in serum and at the plasma cell level in the intestine^11^ in response to dietary gluten suggests that the production of these antibodies is regulated in a coordinated way.

The epitopes recognized by gluten-reactive CD4^+^ T cells of CD patients are well characterized. Typically, the T-cell epitopes harbour glutamate residues, which have been introduced by TG2-mediated deamidation of certain glutamine residues^12^. Less is known about gluten B-cell epitopes. Some epitopes have been characterized by studying polyclonal serum antibody reactivity to synthetic peptides of gliadin proteins^13^ and by bacterial cell-displayed peptide libraries^14^. Deamidation is also relevant for the B-cell epitopes, and serum antibody reactivity is higher to deamidated than the corresponding native (non-deamidated) peptides^13^,^15^.

Gliadin B-cell epitopes appear to be situated in proximity and/or to overlap with gliadin T-cell epitopes^13^,^15^. Furthermore, serum IgA antibodies to TG2, and likely also to deamidated gliadin, only occur in subjects who are HLA-DQ2 or HLA-DQ8 (ref. 16). The HLA dependence of the antibody production and the colocalization of T-cell epitopes and B-cell epitopes suggest that the antibody response to gluten in CD is T cell dependent. Similarly, the HLA dependence of TG2 antibodies suggests T-cell involvement for their formation. Thus, it was surprising that the TG2 IgA expression cloned from CD lesions displayed limited somatic hypermutation (SHM)^5^.

In order to shed further light on the humoral immunity in CD, we have characterized the gluten-specific IgA antibody response of CD that happens in parallel to the autoantibody response to TG2. We have pursued two complementary approaches to identify gliadin-specific IgA^+^ PCs from small intestinal biopsies of subjects with untreated CD (UCD). In one, we isolated gliadin-specific PCs by culturing single PCs and screening the culture supernatants for IgA reactivity to complex gliadin antigen in an epitope unbiased fashion. In another, we analysed and sorted single IgA^+^ PCs with complexes of fluorescent streptavidin and biotinylated synthetic gliadin peptides by flow cytometry. We generated a panel of human monoclonal antibodies (hmAbs) from single PCs specific for gliadin and report that these antibodies have restricted VH and VL usage and limited SHM. These results give insights into the mechanisms of the production of gluten-specific and TG2-specific IgA antibodies in CD and suggest that the limited somatic mutation in both populations is controlled by similar factor(s).

## Results

### Intestinal PCs secreting IgA reactive with gliadin

**Figure 1:**
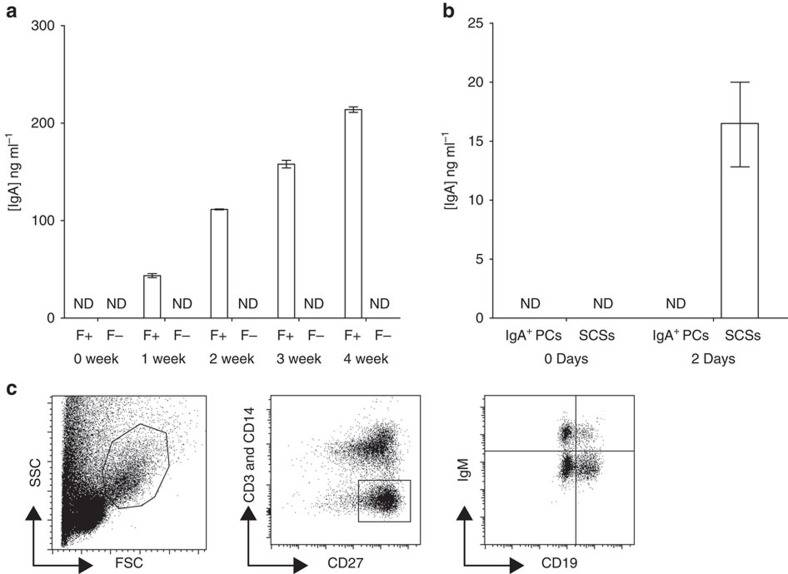
Survival of intestinal PCs in culture. (**a**) Concentration of IgA in supernatants after 0, 1, 2, 3 and 4 weeks culture of SCSs (4-5000 cells ml^−1^) grown with (F+) or without fibroblasts (F−). (**b**) Concentration of IgA after 0 and 2 days in supernatants from cultures of fibroblasts and PCs when PCs were added either as isolated IgA^+^ PCs (IgA^+^ PCs) or as part of SCSs (80-100 IgA+PCs ml^−1^). IgA was detectable only in cultures of PCs as part of SCSs. The bars represents mean values of three separate culture wells and error bars represent s.e.m. (ND denotes non-detectable). (**c**) Representative flow cytometry plots of SCSs after 4 weeks of co-culture with fibroblasts. Viable PCs identified as large (left), viable CD27^+^CD3^−^CD14^−^ (middle) cells as indicated by gating strategy. The PC population divides into IgM^+^ and IgM^−^ PCs, where the IgM^−^ are mainly IgA^+^ PCs (right).

*In vitro* culture was a critical step for isolation of PCs producing antibodies reactive with heat/acid-treated chymotrypsin-digested gliadin (hereafter termed CT-gliadin for short). Three conditions of PC cultures were compared. Single-cell suspensions (SCSs) from intestinal biopsies were generated, and PCs were cultured either as SCSs, as SCSs in co-culture with human intestinal fibroblasts or as PCs isolated by flow cytometry in co-culture with fibroblasts. The concentration of IgA in supernatants of cultures with SCSs together with fibroblasts increased with a constant rate over at least 4 weeks (Fig. 1a), suggesting that the majority of PCs survived in these cultures. We did not observe any increase of IgA in supernatants of cultures of SCSs alone (Fig. 1a) or flow cytometry sorted PCs cultured with fibroblasts (Fig. 1b). In co-cultures of SCSs and fibroblasts, we observed that intestinal PCs survived for weeks, and viable CD19^+^CD27^+^ PCs were detected by flow cytometry after 4 weeks of culture (Fig. 1c). PCs did not proliferate in these cultures, as tested in a BrdU incorporation assay. Based on these findings, the system of co-culturing SCSs and fibroblasts was selected for further experiments.

**Figure 2:**
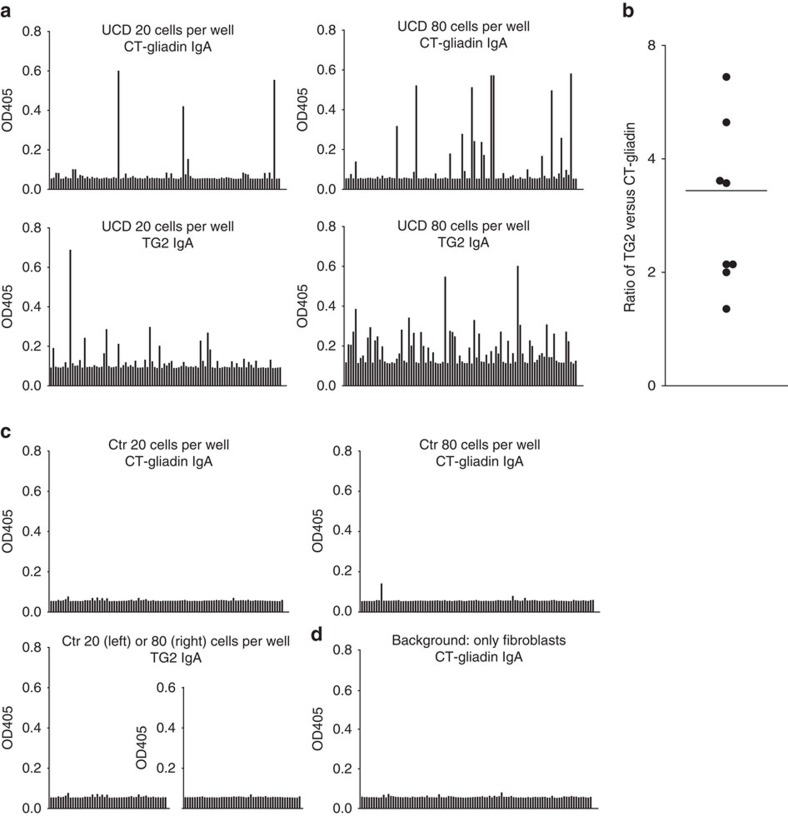
Supernatant reactivity to CT-gliadin and TG2 by ELISA. IgA reactivity to antigen in representative cultures of SCSs from (**a**) subjects with UCD. (**b**) The ratio of culture supernatants with IgA reactivity to TG2 versus CT-gliadin, where the dots represent different subjects with UCD (*n*=8). Horizontal bar indicates mean value. (**c**) IgA reactivity to antigen in one representative of two tested non-CD controls (Ctr). (**d**) The background level was defined by signal in supernatants of cultures of fibroblasts only. In the headline are shown the number of cells of SCSs per culture well and antigen used as coating antigen in ELISA. Each column represents one well.

We aimed at analysing the specificity of antibodies produced at a single PC level. Based on the frequency of IgA^+^ PCs in SCSs that was determined by flow cytometry (mean 3.3%, range 1.0–5.3%, *n*=16) (Supplementary Fig. 1a,b), we seeded 10–20 cells per well to obtain cultures with less than one IgA^+^ PC per well. Supernatants of single wells were screened for IgA reactivity to CT-gliadin and TG2. Supernatants containing IgA reactive to TG2 were three to four times more frequent than IgA reactive to CT-gliadin in cultures from subjects with UCD (Fig. 2a,b). Importantly, IgA reactivity to TG2 and CT-gliadin was not detected in supernatants from cultures of non-coeliac controls (Fig. 2c) or supernatants of cultures containing only fibroblasts (Fig. 2d). Thus, PCs secreting IgA reactive to CT-gliadin are present in small intestinal biopsies of subjects with UCD and are less frequent than PCs secreting TG2-reactive IgA.

**Figure 3:**
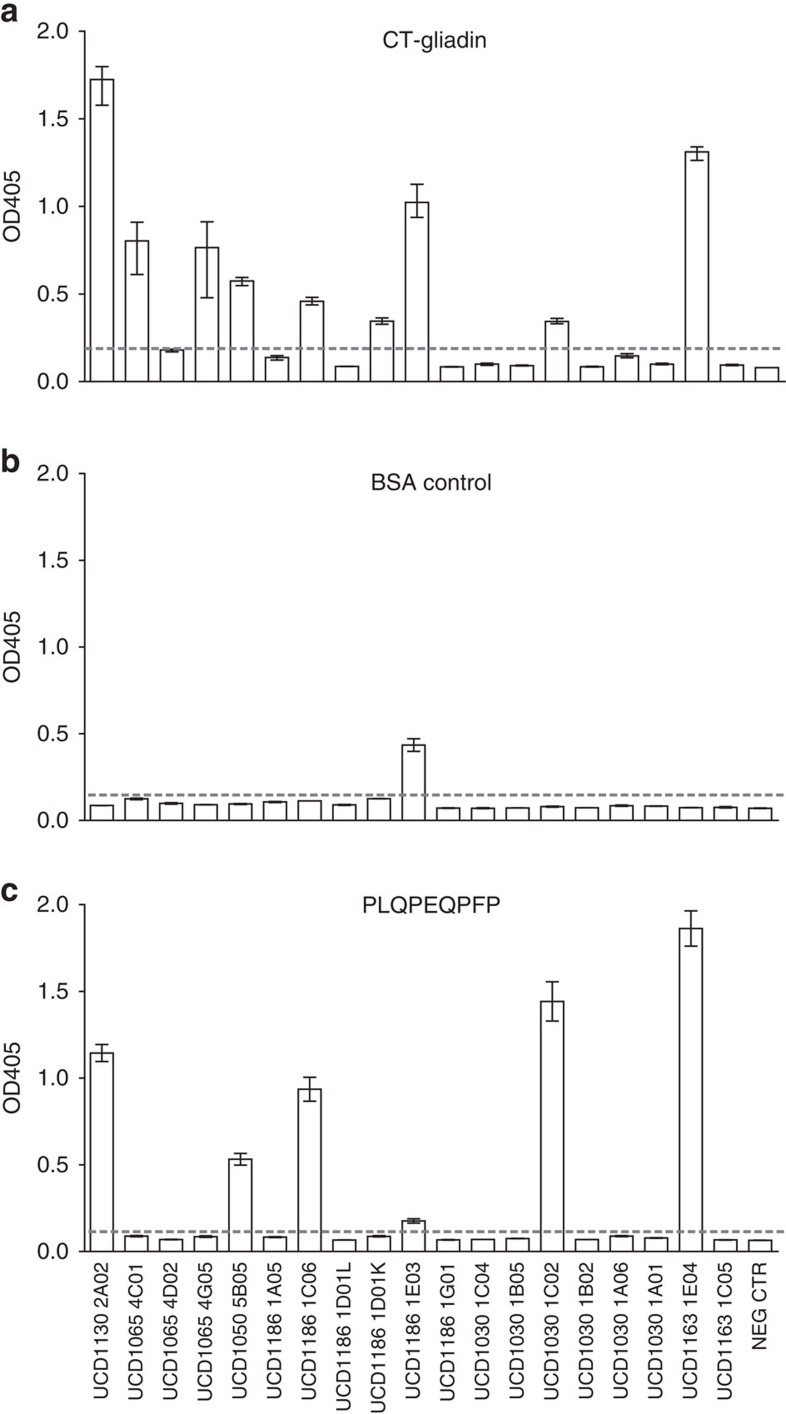
ELISA reactivity of 19 hmAbs expression cloned from IgA^+^ PCs in culture. The antigens used for ELISA coating were CT-gliadin and PLQPEQPFP, and BSA for control as shown in headlines. Values are given as median and range of triplicates of hmAbs tested. The same hmAbs are tested for reactivity to all three antigens, and each hmAb is given the same position in each of the three graphs. The names of the hmAbs are depicted in 3**c**. Horizontal bars denote cutoff defined by negative control hmAb (NEG CTR). (**a**) Nine of 19 hmAbs were reactive to CT-gliadin. (**b**) One of nine hmAbs reactive to CT-gliadin was also reactive to BSA. (**c**) Five of eight hmAbs reactive to CT-gliadin but not BSA were also reactive to PLQPEQPFP.

Next, we wanted to clone and express the antibody genes of PCs from cultures in which we detected IgA reactivity to CT-gliadin in the supernatant. In the SCSs, the large PCs hugely outnumbered small resting B cells (Supplementary Fig. 1c,d). Conceivably, if one well of SCSs contained just one IgA^+^ PC, there was a high probability that this was the only cell expressing the IgA-encoding genes in that well. Where ELISA results suggested gliadin-specific PCs to be present (Fig. 2a), the cells were split into four PCR wells and processed individually to increase the likelihood of having only one PC in each well. Cells were then washed in phosphate-buffered saline (PBS) before snap freezing in DNase-containing buffer and subjected to expression cloning of antibodies in a human IgG1 format as previously described^17^. hmAbs were successfully produced from more than half of cultures processed. Of a total of 19 hmAbs produced, nine were reactive to CT-gliadin (Fig. 3a). These were tested for reactivity to bovine serum albumin (BSA) and CpG, of which one was found reactive to BSA (Fig. 3b). Thus, eight hmAbs were considered to be gliadin specific and included in further assays.

### Intestinal IgA^+^ PCs stained with gliadin peptides in flow cytometry

We had previously succeeded in visualizing antigen-specific IgA^+^ PCs by staining with biotinylated antigen bound to fluorescent streptavidin taking advantage of surface IgA and IgM expression of gut PCs^5^,^18^. We used the same strategy to identify gliadin-specific IgA^+^ PCs by flow cytometry, using fluorescent streptavidin to form tetramer complexes with biotinylated synthetic peptides. Two different gliadin peptides with sequences targeted by serum IgA antibodies of CD patients, biotin-(GSGSGS)-PLQP**E**QPFP^13^ and biotin-(PEG)-LQLQPFPQP**E**LPYPQP**E**LPYPQP**E**LPYPQPQPF^19^, were used as antigen. The two peptides represent sequences of gliadin with Q to E transitions in positions targeted by TG2 (ref. 12). Importantly, both peptides are reported to bind antibodies from UCD patients, primarily in their deamidated versions^15^. PLQP**E**QPFP in particular has demonstrated high sensitivity and specificity for CD in serologic assays^20^. This approach using synthetic peptides, introduced a restricted selection of gliadin-specific PCs compared with screening culture supernatants for IgA reactivity against CT-gliadin.

**Figure 4:**
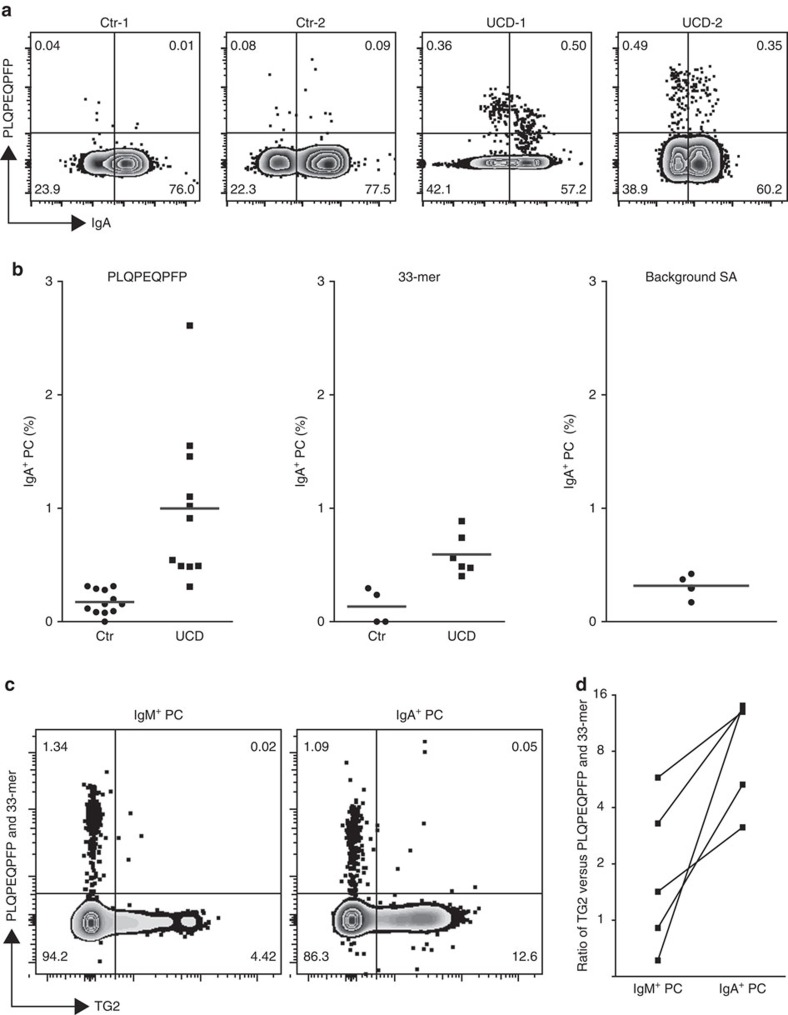
Staining of intestinal PCs with tetramers of synthetic gluten peptides or TG2 in flow cytometry. (**a**) Representative plots of PCs from SCSs stained with APC-conjugated streptavidin in complex with biotinylated PLQPEQPFP peptide. The IgA-negative events are mainly IgM^+^ PCs. The four plots represent two control patients (Ctr) and two subjects with UCD. (**b**) Frequency of IgA^+^ PCs stained positive with peptide in percentage of total number of IgA^+^ PCs. The peptide used as antigen is given in headline. Each dot represents one subject, and mean values are indicated by horizontal bars. ‘Background SA’ represents cells stained with APC-streptavidin alone. (**c**) Representative plots of one subject with UCD of IgM^+^ PCs and IgA^+^ PCs from SCSs stained with biotinylated peptides in complex with APC-conjugated strepavidin and biotinylated TG2 in complex with PE-conjugated streptavidin. (**d**) Ratio of PCs stained positive with TG2 compared with synthetic gliadin peptides. IgM^+^ PCs and IgA^+^ PCs of the same sample are connected by lines.

In flow cytometry, the mean percentage of IgA^+^ PCs stained with the PLQPEQPFP peptide tetramers was 1.0% (range 0.3–2.6%, *n*=10) of total IgA^+^ PCs in SCSs generated from biopsies taken from UCD patients (Fig. 4a,b). In comparison, the mean percentage in non-CD disease controls was 0.2% (range 0–0.3%) (Fig. 4a,b). For IgA^+^ PCs stained with the deamidated 33-mer peptide tetramers, the mean percentage was 0.5% (range 0.4–0.9%, *n*=6) for subjects with UCD and 0.1% in controls (range 0–0.3%, *n*=4). Background levels were defined by staining IgA^+^ PCs with fluorescent streptavidin alone (mean 0.3%, range 0.2–0.4%, *n*=4) (Fig. 4b).

Importantly, IgA^+^ PCs stained with synthetic gliadin peptides and TG2 appeared as two separate populations in flow cytometry whereas double-positive cells were not detected (Fig. 4c). Also, the populations visualized with TG2 were larger than the populations stained with both the PLQPEQPFP and the deamidated 33-mer peptides (Fig. 4d). These findings are consistent with the ratio of the frequencies of IgA^+^ PCs reactive with TG2 versus those reactive with CT-gliadin observed in culture (Fig. 2b). The predominance of TG2^+^ PCs relative to the population stained with gliadin peptides was greater for the IgA^+^ PCs than for the IgM^+^ PCs (Fig. 4d). Based on these experiments, we conclude that there are two distinct populations of gliadin-specific and TG2-specific IgA^+^ PCs in the small intestinal lesions of UCD patients.

IgA^+^ PCs stained with fluorescent streptavidin in complex with either PLQPEQPFP or deamidated 33-mer were isolated as single cells by flow cytometry for further expression cloning. The hmAbs obtained were tested for reactivity to the peptide, which they were originally isolated with. Seventeen of 23 hmAbs from PCs isolated with the PLQPEQPFP peptide, and 13 of 16 hmAbs from IgA^+^ PCs isolated with the deamidated 33-mer peptide were reactive in ELISA to the peptide originally used in sorting. This indicates that the staining specificity of gluten peptide tetramers was ~75–80%.

### Affinity of gliadin-specific hmAbs to deamidated and native gliadin

The eight hmAbs that originated from *in vitro* PCs cultures reactive with CT-gliadin were tested in ELISA against the two synthetic peptides. Five reacted with PLQPEQPFP (Fig. 3c) and one reacted with deamidated 33-mer. Hence, six out of eight hmAbs were reactive to PLQPEQPFP and/or deamidated 33-mer. Since cloning of antibody genes from those cultures is unbiased with respect to specific epitope, this result suggests that the epitopes represented by PLQPEQPFP and the deamidated 33-mer are recognized by IgA of a significant part of gliadin-specific PCs.

The specificity of all hmAbs reactive to one of the two synthetic peptides in ELISA was further characterized in AlphaLISA. In contrast to ELISA, where synthetic peptide was used as a coating antigen, the AlphaLISA format detected monovalent binding of hmAbs to soluble biotinylated synthetic peptide. Three of the hmAbs (UCD1002 1D03, UCD1143 1E01, UCD1130 4A04), originally from IgA^+^ PC sorted with tetramers of PLQPEQPFP and reactive to PLQPEQPFP in ELISA, were not reactive in AlphaLISA. One of the hmAbs (UCD1130 3B04) from IgA^+^ PC sorted with 33-mer and reactive to deamidated 33-mer in ELISA was not reactive in AlphaLISA. These four antibodies, not reactive in AlphaLISA, gave the lowest signals in the peptide-based ELISA and with signals that were lower than to the CT-gliadin antigen (Supplementary Fig. 2), suggesting that the synthetic peptides may not represent the complete epitope(s) of these four antibodies.

**Figure 5:**
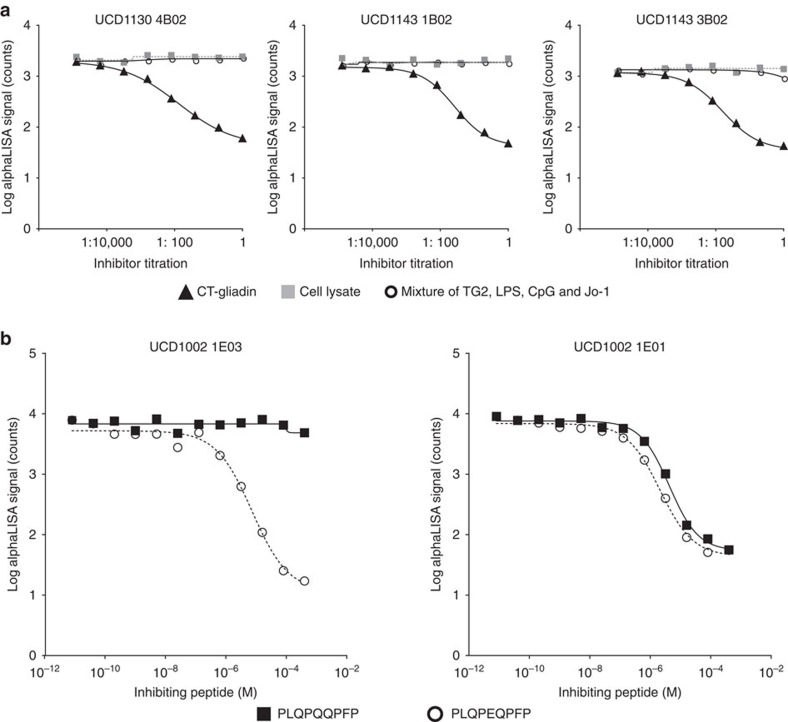
AlphaLISA characterization of gliadin-reactive hmAbs. (**a**) Antibody reactivity to constant concentration of biotinylated PLQPEQPFP in the presence of competing antigens. The starting concentrations of competing antigens are CT-gliadin 10 μg ml^−1^, cell lysate 1 ml cells ml^−1^, TG2 200 μg ml^−1^, LPS 250 μg ml^−1^, CpG 100 μg ml^−1^ and Jo-1 200 μg ml^−1^. Only CT-gliadin blocked the signal, while cell lysate or a mixture of TG2, LPS, CpG and Jo-1 did not. (**b**) Reactivity to deamidated gliadin versus native gliadin. Representative plots of two hmAbs binding to constant concentration of biotinylated PLQPEQPFP in competition with various concentrations native PLQPQQPFP or the deamidated PLQPEQPFP was measured. The concentrations of competitive peptide are depicted on *x* axis.

Polyreactivity was assessed by using cell lysate as well as a mixture of Jo-1, TG2, CpG and LPS as competing antigens. In either case, the binding of hmAbs to the synthetic peptides was maintained (Fig. 5a). In contrast, the signal was blocked by CT-gliadin (Fig. 5a). We conclude that the hmAbs reactive to PLQPEQPFP or deamidated 33-mer are specific to gliadin and not polyreactive.

The reactivity of hmAbs to deamidated versus native gliadin was tested in a competitive AlphaLISA assay. Binding of hmAbs to biotinylated synthetic deamidated gliadin peptide (either PLQPEQPFP or the deamidated 33-mer) was tested against the homologous peptide in either deamidated or native (non-deamidated) versions. Gliadin-specific hmAbs divided into two groups, either only reactive to deamidated gliadin or reactive with both deamidated and native gliadin (Fig. 5b). None of the hmAbs had higher reactivity to native than to deamidated gliadin. Because some of the hmAbs were not reactive to the native gliadin peptide, this suggests that induction of gliadin-specific B cells *in vivo* likely happens in the presence of deamidated gliadin.

### Gliadin-specific hmAbs bind gliadin peptides with shared sequences

**Figure 6:**
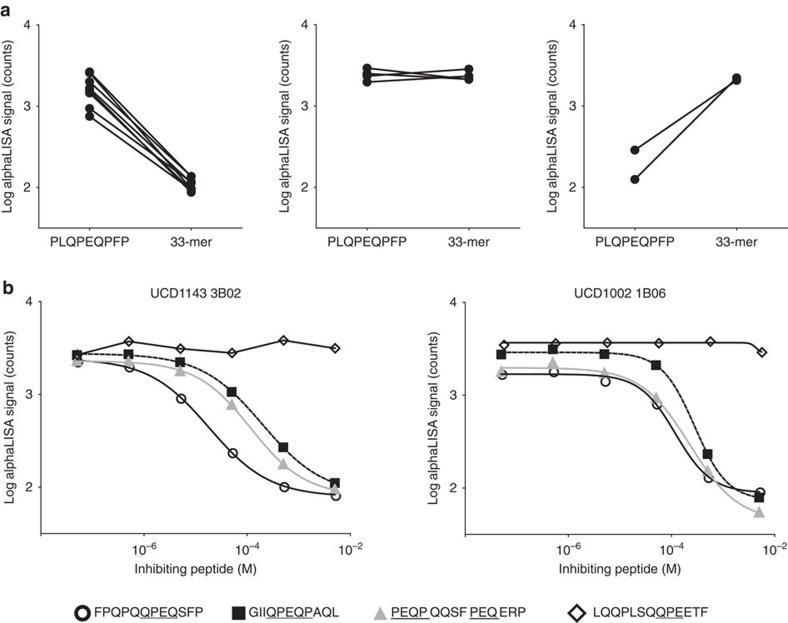
Epitopes of gliadin-specific hmAbs. (**a**) Reactivity of hmAbs to PLQPEQPFP and 33-mer. Reactivity of each hmAb is connected by line. Antibodies are grouped according to their reactivity pattern with reactivity to only one of the two peptides (left, right) or cross-reactive with both peptides (middle). (**b**) Reactivity of two representative hmAbs to constant concentration of biotinylated PLQPEQPFP in competition with four different competitive synthetic gliadin peptides. The amino-acid sequences of competitive peptides are shown and the concentrations of competitive peptides are depicted on *x* axis. Amino-acid sequences shared with the PLQPEQPFP selecting peptide are underlined.

We observed that some of the hmAbs were reactive with both PLQPEQPFP and the deamidated 33-mer in AlphaLISA (Fig. 6a). Eight of the hmAbs originally from IgA^+^ PC sorted with tetramers of PLQPEQPFP were reactive to deamidated 33-mer (Supplementary Table 1). Of the hmAbs from IgA^+^ PC sorted with deamidated 33-mer, eleven were reactive to PLQPEQPFP (Supplementary Table 1). This suggests that at least some of the gliadin-specific PCs secrete antibodies that cross-react with different gliadin peptides. To test this further, we investigated whether a few selected synthetic gliadin peptides could compete with the antibody binding to biotinylated PLQPEQPFP. We tested the hmAbs (UCD1143 3B02 and UCD1002 1B06) both of which were reactive with PLQPEQPFP but not PLQPQQPFP (Supplementary Table 1). For both hmAbs, the signals were inhibited by peptides containing QPEQ or PEQP. Peptides without these sequences were non-inhibitory (Fig. 6b). Thus, the cross-reactivity of hmAbs to different gliadin peptides appears to be due to sharing of key sequences. Reactivity of many hmAbs to PLQPEQPFP and deamidated 33-mer, both harbouring the QPEXP (X=Q, L) and QPFP sequences, is consistent with this notion.

### Binding to PLQPEQPFP of hmAbs is blocked by patient sera

**Figure 7:**
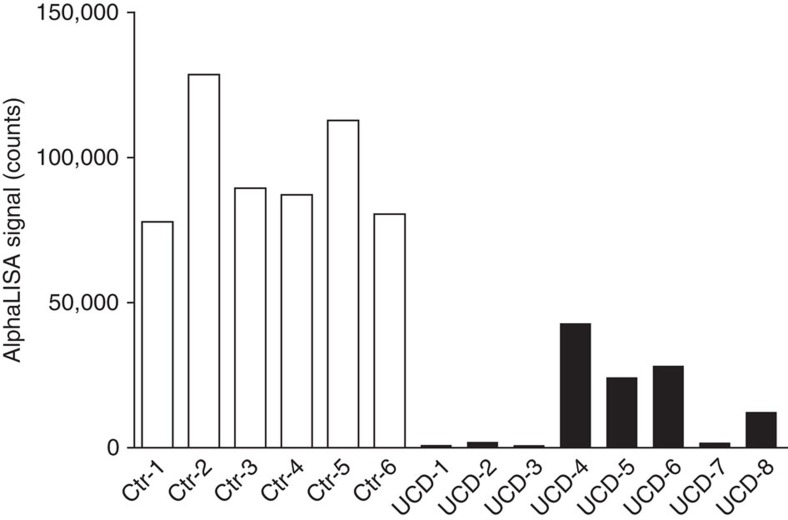
AlphaLISA anti-PLQPEQPFP immunoglobulin inhibition assay. Reactivity of hmAbs UCD1114 1F03 and UCD1143 3B02 to constant concentration of biotinylated PLQPEQPFP in the presence of sera from either UCD or control subjects (Ctr). The signal was blocked by sera from UCD patients but not from control subjects.

We wanted to see if binding of gliadin-specific hmAbs to synthetic gluten peptide was blocked by sera from patients with active CD. In a preliminary study, sera were incubated with biotinylated PLQPEQPFP before incubation with AlphaLISA beads conjugated with two hmAbs UCD1114 1F03 and UCD1143 3B02. Sera from patients with CD blocked the signal, while sera from control subjects did not (Fig. 7). This suggests that gliadin-specific antibodies from serum and IgA of gliadin-specific PCs in the lamina propria are specific to the same gliadin B-cell epitopes. This is also a promising result as a proof of concept that hmAbs derived from antigen-specific human B cells can be used to establish improved serologic assays.

### Repertoire of gliadin-specific IgA^+^ PCs

**Figure 8:**
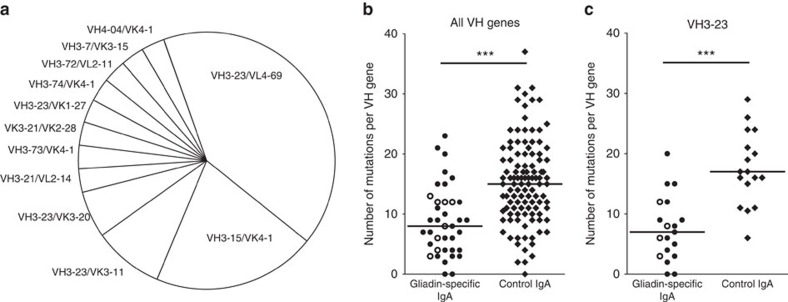
VH and VL usage and SHMs. (**a**) VH/VL usage of 38 gliadin-specific hmAbs from PCs either isolated by culture or by flow sorting. (**b**) Frequencies of VH region somatic mutations (*y* axis) per sequence in the populations indicated on *x* axis. ‘Gliadin-specific IgA’ represents hmAbs from PCs sequestered after *in vitro* culture (open circles) and IgA^+^ PCs isolated with synthetic gliadin peptides (black dots). ‘Control IgA’ represents sequences derived from intestinal IgA^+^ PCs sorted as TG2-negative and PLQPEQPFP/33-mer-negative from two subjects with untreated CD (UCD1130 and UCD1143). Each point represents a single IgA sequence. (**c**) Comparison of mutations in VH3-23 genes. Horizontal bars indicate median values and *P*-values were obtained by Student’s *t*-test. NS>0.05. ***P<0.001.

The eight hmAbs from *in vitro*-cultured PCs with CT-gliadin were obtained from six subjects (UCD1050, 1; UCD1130, 1; UCD1065, 2; UCD1186, 2; UCD1163, 1; UCD1030, 1). The 30 hmAbs derived from single-sorted PCs using tetramerized synthetic peptides, originated from five different subjects (UCD1002, 7; UCD1079, 2; UCD1114, 2; UCD1130, 16; UCD1143, 3). Control IgA^+^ PCs, defined as PLQPEQPFP-negative, 33-mer-negative and TG2-negative (UCD1130, 55; UCD1143, 54) and TG2-specific PCs (UCD1030, 3; UCD1114, 4; UCD1010, 2), were isolated by flow cytometry and their antibody genes were cloned and sequenced. The variable regions of the hmAbs were analysed using tools of the IMGT webpage (www.imgt.org). Two combinations of VH/VL pairing were dominant (Fig. 8a); VH3-15/VK4-1 (seven hmAbs from five different subjects in total) and VH3-23/VL4-69 (15 hmAbs from seven different subjects in total). The VH3-23/VL4-69 combination was found in hmAbs from IgA^+^ PCs isolated with both CT-gliadin and synthetic peptides. In addition, VH3-23/VK3-11 was also found in hmAbs generated by both methods. Together, these three combinations made up ~75% of the panel of gliadin-specific hmAbs (Fig. 8a, Supplementary Table 1). We looked for similarity between heavy chain CDR3 sequences of the VH3-15/VK4-1 as well as the VH3-23/VL4-69 hmAbs. No obvious similarities could be found with variability in D- and J-gene segment usage and CDR3-length among the antibodies of each group (Supplementary Table 1).

### VH/VL usage and epitope specificity

We next compared VH/VL usage (Supplementary Table 1) with peptide reactivity pattern characterized in AlphaLISA. All VH3-23/VL4-69 hmAbs showed reactivity to both native and deamidated gliadin, demonstrated by inhibiting effect of both the native PLQPQQPFP and the deamidated PLQPEQPFP. The VH3-15/VK4-1 hmAbs were specific to deamidated gliadin, as the native PLQPQQPFP showed no inhibitory effect on the binding to PLQPEQPFP. VH3-23/VK3-11 hmAbs were the only antibodies with reactivity to deamidated 33-mer but not PLQPEQPFP. Two hmAbs had reactivity to CT-gliadin but neither to PLQPEQPFP nor to deamidated 33-mer. These two antibodies both used VH3-15/VK3-20. Taken together, this suggested that there is a correlation between VH/VL usage and epitope specificity of the gliadin-specific antibodies.

### SHM within gliadin-specific IgA^+^ PCs

The mutation rates in the hmAbs from PCs secreting IgA reactive with CT-gliadin (median 8.7, range 3–13, *n*=8) as well as IgA^+^ PCs isolated with synthetic gliadin peptides (median 7.5, range 0–23, *n*=30) were lower than in the control PCs (median 15, range 0–37, *n*=109) (Fig. 8b). TG2-specific IgA^+^ PCs were scarcely mutated as previously described^5^ (median 4, range 0–15, *n*=9).

Different VH genes are associated with various levels of SHM^21^. The mutation rate in gliadin-specific IgA with VH3-23 was significantly lower than the number of mutations observed in VH3-23 from control population (Fig. 8c). We conclude that the low mutation rate in gliadin-specific IgA is not a result of selected VH usage.

## Discussion

We previously reported that IgA antibodies of TG2-specific PCs found in coeliac lesions have limited SHM^5^. In the present study, we have characterized gluten (gliadin)-reactive antibodies expression cloned from IgA^+^ PCs isolated from small intestinal biopsies. We found that there is also a limited mutation rate in these antibodies, demonstrating an unexpected common feature of the IgA antibody responses to gluten and TG2 in CD.

The number of mutations in antibody genes relates to the type of B-cell response elicited^22^. T-cell-independent B-cell responses have no or little SHM with little restriction of VH usage as often seen in T cell-dependent responses^23^. T-cell-dependent B-cell responses that develop in germinal centers typically result in highly mutated antibodies^24^. GC reactions may also happen without the involvement of T cells, but then with low level of SHM^25^. Low level of SHM is also seen in T-cell-dependent extrafollicular responses^26^ and in germinal center responses of short duration^27^.

For the TG2-specific IgA antibodies, the low mutation rate was suggested to relate to enzymatic activity of B-cell receptor bound TG2 (ref. 5) or to the early termination of autoreactive B cells in GCs or self-antigen driving an extrafollicular response, similar to what has been reported in other systems^22^,^28^,^29^. None of these mechanisms should apply to IgA antibodies of gluten-specific PCs, and one could therefore expect that these PCs should have similar levels of SHM compared with control PCs. This is not what we observed. The mutation rate in IgA of gluten-specific PCs was low and similar to TG2-specific PCs. This suggests that the factor(s) causing the limited SHM in IgA of TG2-specific PCs is likely involved in both antibody responses.

The parallel fluctuation of antibodies to gluten and TG2 in response to dietary gluten^10^ suggests that the production of these antibodies is regulated in a coordinated way. In keeping with this notion, it has been suggested that gluten-specific T cells could provide help to TG2-specific B cells by means of complexes gluten-TG2 acting as hapten-carrier complexes^30^,^31^. This model would explain why the TG2 antibodies are produced upon gluten exposure, and it would also explain why only individuals who express HLA-DQ2 or HLA-DQ8 make these antibodies^16^. Gluten-specific T cells obviously also could provide help to gluten-specific B cells. As gluten-specific T cells preferentially recognize deamidated gluten peptides^32^, B cells with surface immunoglobulin that bind and internalize deamidated gluten peptides would be better situated to receive T-cell help. Common sequences of the two gliadin peptides used to isolate IgA^+^ PCs, are found in or adjacent to many T-cell epitopes in gluten proteins. The QPEXP (X=Q, L) sequence is found in the DQ2.5-glia-α1a/b, DQ2.5-glia-α2, DQ2.5-glia-γ2, DQ2.5-glia-γ3, DQ2.5-glia-γ4c/d, DQ2.5-glia-φ1, DQ2.5-glia-φ2, DQ2.5-hor-1, DQ2.5-hor-2, DQ2.5-sec-1, DQ2.5-sec-2 and DQ8-glia-γ1a/b epitopes whereas the QPFP sequence is found in the DQ2.5-glia-γ4c, DQ2.5-glia-γ5, DQ2.5-glia-φ2, DQ2.5-hor-2, DQ2.5-sec-2 and DQ8-glia-γ1a epitopes^32^. The low mutation rate in TG2- and gluten-specific PCs compared with other intestinal PCs, suggests that a common factor distinguishing the TG2- and gluten-specific PCs from most other gut PCs could be implicated. Based on the model referred above, both gluten and gluten-reactive T cells could be such common factors.

Gluten is a protein antigen, which in contrast to most antigens of the gut has no bacterial or viral origin. Lack of strong concomitant innate signals along with B-cell receptor triggering could result in extrafollicular response, in accordance with a study of the B-cell response to a T cell-dependent antigen, where immunization without adjuvant was insufficient for GC formation^33^. Analysis of mutation rate of antibodies to other dietary proteins would shed light on this and should be examined in the future.

Extrafollicular responses typically give rise to short-lived PCs and rapid decline in serum antibodies after the immune response in addition to low SHM^34^. TG2-specific PCs were rarely detected in small intestine of CD subjects on a gluten-free diet^5^, and levels of serum IgG and IgA antibodies to both gluten and TG2 typically fall below detection level months after the patient commence a gluten-free diet^10^. These clinical observations, in addition to our observations of limited SHM in gluten-specific and TG2-specific IgA^+^ PCs, argue for an extrafollicular origin of gluten and TG2-specific antibodies in CD.

T cells influence extrafollicular development of B cells. It has been reported that T cells located at the T-B border in lymph nodes, phenotypically different from GC T cells, are necessary for B-cell priming to form extrafollicular antibody responses^35^. Sustained CD40 signalling during B-cell and T-cell interaction has been demonstrated to induce a plasma cell fate rather than GC B-cell development^36^, and administration of a CD40 agonistic antibody was shown to ablate GC reaction and induced a pattern of extrafollicular B-cell differentiation^37^.

Not only the quality, but also the degree of T-cell help could be a relevant factor. Excessive numbers of follicular helper T cells as seen in several mouse models, like the ‘san’ mutation in the RNA binding protein Roquin^38^ and PD-1 deficiency^39^, drive survival of GC B cells with a low degree of mutation and affinity and cause increased autoreactivity possibly due to a lowered threshold for GC B-cell selection^40^. Interestingly, gluten-reactive T cells of CD patients produce large amounts of interferon-γ^41^ and in mice, excess of interferon-γ has been demonstrated to promote accumulation of both follicular helper T cells and GC B cells and to trigger autoimmunity^42^. It may thus be that TG2- and gluten-reactive B cells in such a setting may develop in GC and not extrafollicularly.

Characterization of the specificity of antibodies produced by PCs reactive with gluten should give valuable insights into the pathogenesis in CD. We thus characterized the panel of hmAbs by AlphaLISA assays. First, we found that some antibodies are specific to deamidated gliadin peptides and do not recognize non-deamidated counterparts. Inevitably, gliadin-specific B cells must have encountered deamidated gliadin. Second, we found that gliadin-specific antibodies frequently cross-react with different gliadin peptides. Accordingly, gliadin-specific B cells may take up and display several different T-cell epitopes. This may be beneficial if a B cell receives help from several different T-cell clones, as recently described^43^.

In addition to the limited SHM, we observed a restricted VH/VL usage in the panel of gluten-specific antibodies. Limited VH usage is a sign of T-cell-dependent responses^23^. Notably, the same VH/VL pairings appear in different subjects. This phenomenon is similarly seen in TG2-specific antibodies where VH restriction is observed in different patients^5^. The VH usage of TG2-specific antibodies was recently demonstrated to correlate with epitope specificity^44^. We observed a similar phenomenon for the gliadin-specific hmAbs. Antibodies specific to deamidated variants of gluten peptides seemed to have different VH/VL usage than antibodies with reactivity to both the deamidated and native variants. Such VH/VL restriction is by no means unique to CD and has been reported in antibodies specific for influenza^45^ and HIV^46^ antigens. For HIV antibodies, there has even been reported an allelic preference for antibody usage^46^. A deeper analysis may reveal similar allelic preferences of the antibody responses to TG2 and gluten in CD. Restricted VH/VL usage and limited SHM in antibodies as observed in CD would favour influence of VH and/or VL polymorphisms in shaping the antibody response. This could speak for the involvement of VH and VL genes as susceptibility loci in CD, even though these genes have not been indicated by recent genome-wide association studies in humans^47^. Notably in mice, experiments with congenic strains demonstrated that the antibody response to gliadin is chiefly controlled by the MHC and the immunoglobulin heavy chain loci^48^.

Interestingly, the hmAbs generated can be used to develop diagnostic assays detecting patient serum antibodies reactive with disease-relevant gluten epitopes. Given that the antibodies are derived from PCs of the CD lesion, inhibition assays reflecting the specificity of the hmAbs may potentially perform better than assays used today. The hmAbs could potentially also be used for detection of gluten contamination in gluten-free food.

In summary, our findings of restricted mutation rates in both IgA antibody responses to gluten and TG2 in CD are surprising. We suggest that a common factor important for gluten-specific and TG2-specific B cells, possibly gluten antigen or gluten-specific T cells, is responsible for causing low mutation rates in the specific IgA antibodies. There is a restricted, but different usage of VH and VL gene segments, for recognition of gluten and TG2 in CD. The restricted usage raises the possibility that there might be genetic effects at the immunoglobulin loci for the development of this disease.

## Methods

### Subjects

Small intestinal biopsy specimens were obtained from subjects by esophagogastroduodenoscopy and forceps sampling from the duodenum. UCD patients were referred to endoscopy based on clinical suspicion and positive serologic test, and diagnosed according to the guidelines of the American Gastroenterological Association Institute^49^. These subjects were still on a gluten-containing diet at the time when biopsies were obtained, that is, when the disease was in its active chronic stage. Non-CD controls were appointed to endoscopy, where histopathology and serology negated CD diagnosis. The non-CD control group consisted of seven HLA-DQ2.5+ subjects, three HLA-DQ8+ subjects and four subjects who were not HLA-typed. All subjects have given written informed consent. The study was approved by Regional Committees for Medical Research Ethics South East Norway (S-97201).

### Single-cell suspensions

Intestinal biopsies were collected and transported in RPMI 1640 in 50 ml tubes on ice. For preparation of SCSs^18^, the biopsies were transferred to 15 ml tube and resuspended in 2 mM EDTA in 2% fetal calf serum (FCS) in PBS. After 30-min rotation at 37 °C, the supernatant was discarded and the biopsies resuspended in 1 mg ml^−1^ blend collagenase (Sigma, C8051) and 50 μg ml^−1^ DNase (Sigma, DN25) in 2% FCS in Dulbecco’s PBS. The biopsies were then incubated under constant rotation at 37 °C. After 30 min, the biopsies were mechanically disrupted with a syringe equipped with a large steel needle. After another 30 min constant rotation, a smaller needle was used for the same procedure. After 1–2 h, the single-cell suspension was filtered through 40 μM filter into 50 ml tube and centrifuged at 470 *g* for 7 min.

### Human intestinal stromal cell line

Human fibroblast cell lines were derived from small intestinal biopsies as previously described^50^. Biopsies were transferred to flat-bottomed six-well plates and gently disrupted with a scalpel for 15–30 s. The biopsies were cultured in 1% penicillin/streptomycin in 10% FCS in RPMI 1640 (culture medium) at 37 °C in 5% CO_2_. Medium was changed every second week. After 5–10 weeks, a dense layer of cells attached to the bottom was detectable in the wells. These cells were detached with 0.05% trypsin-EDTA (Gibco, 25300–054) and transferred to 25 ml culture flasks. Culture medium was changed regularly. In these flasks, the cell lines typically survived for 6–9 months. One of these cell lines, F1100 from subject UCD1100, was used in most of the experiments.

### *In vitro* co-culture of SCSs and fibroblasts

First, fibroblasts were detached from 25 ml culture flasks and transferred to plates with flat-bottomed wells. After 1 week, cells from SCSs were seeded on confluent layer of fibroblasts. Fibroblasts and SCSs were incubated in culture medium at 37 °C in 5% CO_2_. Different plate formats were suited to different experimental settings; 24-well plates were used for BrdU assays and estimation of total IgA production and 384 well plates were used of single PC cultures.

### Extraction and preparation of gliadin proteins

Gliadin, the alcohol soluble component of gluten, was extracted from wheat flour. Wheat flour (Møllerens 550001) 50 g was dissolved in 150 ml butanol, vortex mixed and centrifuged at 163 *g* for 5 min. The butanol was decanted and the procedure repeated. The wheat flour pellet was dissolved in 350 ml 70% ethanol and incubated at RT overnight under constant stir mixing. Next day, the solution was centrifuged at 650 *g* for 5 min, and the supernatant was mixed with 1.5 M NaCl in ratio 1:2 and incubated at 4 °C for 4 h to precipitate the gliadin proteins. After centrifugation at 25,000 *g* for 20 min, the supernatant was decanted and the gliadin pellet dissolved in 40 ml 8 M urea in 0.01 M ammonium bicarbonate. This solution was diluted 1:4 to give a final urea concentration of 2 M and incubated with 12–24 mg chymotrypsin (CT) overnight at 37 °C under constant stir mixing. Next day, chymotrypsin was heat inactivated at 98 °C for 5 min, and the solution was dialyzed (Spectra/Por Membrane MWCO 1,000) overnight and dried in speed vacuum concentrator. The digested gliadin was dissolved and incubated in acetic acid pH 1.8 at 95 °C for 1 h to introduce Q to E conversion (deamidation). The final product, heat/acid-treated chymotrypsin-digested gliadin (CT-gliadin for short), was diluted in distilled H_2_O and freeze dried before further usage.

### ELISA supernatant IgA reactive with CT-gliadin

ELISA plates (96 well Nunc 436014) were coated with 75 μl per well of CT-gliadin 40 μg ml^−1^ in carbonate buffer 0.05 M pH 9.6 overnight at 4 °C, washed and subsequently blocked with 0.5% BSA in PBS and incubated with supernatant from single PC cultures. Anti-human IgA-alkaline phosphatase (Sigma, A9669) 1:3,000 was used as secondary antibody. Anti-human IgG-alkaline phosphatase (Southern Biotech, 2040-04) at concentration 1:4,000 was used when testing gliadin-reactive hmAbs. Plates were developed for ~15 min with phosphatase substrate (Sigma-Aldrich) and absorbance was measured at 405 nm.

### Isolation of PCs after *in vitro* culture

Cells from culture wells were transferred to PCR plate and centrifuged at 3,750 *g* for 5 min. Supernatant was decanted, and RNAse-inhibiting RT–PCR catch buffer^17^ was added, 10 μl per well. PCR plate was sealed and stored at -70 °C until single-cell PCR preparation.

### Staining antigen-specific PCs from SCSs

Two different biotinylated synthetic gliadin peptides were used to stain gliadin-specific PCs from SCSs. Biotin-GSGSGS-PLQPEQPFP and biotin-GSGSGS-LQLQPFPQPELPYPQPELPYPQPELPYPQPQPF were produced by GL Biochem (Shanghai). TG2 was expressed in Sf9 insect cells by Phadia and linked to biotin with Sulfo-NHS-LC-biotin (Pierce) as per the manufacturer’s protocol.

The synthetic peptides were incubated on ice with APC-labelled streptavidin (PhycoLink, PJ27S) and biotinylated TG2 incubated with PE-labelled streptavidin (Invitrogen, S866) at 4:1 molar ratio, in the dark for 1 h. The final tetramer concentration for staining intestinal PCs was 40 nM in staining buffer containing 2% FCS in PBS. PE CD138 1:40 (eBioscience, DL-101), PE-Cy7 CD27 1:50 (eBioscience, LG.7F9), Pacific Blue CD19 1:100 (BioLegend, HIB19), PerCP CD3 1:40 (BD Biosciences, SK7), PerCP CD14 1:40 (BD Biosciences, MφP9), FITC goat anti-human IgA 1:800 (Southern Biotech, 2050-02) were used for staining of SCS. Propidium iodide for exclusion of dead cells was added just before analysis. Three different flow cytometer instruments were used: Facs Aria, LSRII and Fortessa. The plots in this manuscript (Figs 1 and 3, Supplementary Figs 1 and 2) are from LSRII. PCs appeared as one homogeneous population of large, CD4^−^ and CD14^−^ events, coexpressing CD138 and CD27, and were previously identified as antibody producing cells^18^. CD27 stained with higher intensity and gave a more defined population than CD138. Thus, PCs were defined as large, viable, CD27^+^CD3^−^CD14^−^ events.

### Cloning and expression of hmAbs

The variable regions of the heavy and light chain antibody genes of isolated PCs were amplified by RT–PCR and nested PCR using the same primers as previously described^17^, cloned into expression vectors and transfected into a human cell line as IgG1 according to previously established protocol^17^.

### AlphaLISA screening of hmAbs for reactivity to gluten peptides

AlphaLISA Acceptor beads (Perkin Elmer, 6772001) were coupled with polyclonal rabbit anti-human IgG (Dako A0423) and stored at a concentration of 2.5 mg ml^−1^ according to the manufacturer’s instructions (as gliadin-specific hmAbs were produced in human IgG1 format). Anti-IgG AlphaLISA donor bead solution (1:400) and gliadin-specific hmAbs 1 μg ml^−1^ in AlphaLISA immunoassay buffer (Perkin Elmer, AL000C) were incubated for 1 h at 4 °C in the dark. After incubation, 15 μl were transferred to each well in 384 well plates and mixed with 5 μl analyte. The plates were incubated for 1 h at RT in the dark. AlphaScreen streptavidin donor bead solution (Perkin Elmer, 6760002B) was diluted 1:200 in AlphaLISA Immunoassay buffer, and 15 μl were added per well before incubation at RT in the dark for 30 min. The different peptides used in the analyte were the following: biotin-GSGSGS-PLQPEQPFP, PLQPQQPFP, PLQPEQPFP, GIIQPEQPAQL, LQLQPFPQPQLPYPQPQLPYPQPQLPYPQPQPF, FPQPQQPEQSFP, PEQPQQSFPEQERP, LQLQPFPQPELPYPQPELPYPQPELPYPQPQPF, LQQPLSQQPEETF and biotin-GSGSGS-LQLQPFPQPELPYPQPELPYPQPELPYPQPQPF were purchased from GL Biochem, Research Genetics, Neosystems or obtained from Burkhard Fleckenstein.

AlphaLISA Acceptor beads were also coupled to equal amounts of the two gliadin-specific hmAbs UCD1114 1F03 and UCD1143 3B02 and stored at concentration 2.5 mg ml^−1^. These beads were used to detect biotin-GSGSGS-PLQPEQPFP in the presence of serum (Fig. 7). First, 5 μl biotin-GSGSGS-PLQPEQPFP was incubated with 10 μl serum at RT for 30 min. Then, 30 μl of mixture of AlphaLISA acceptor bead solution (1:600) and Alphascreen donor bead solution (1:300) in AlphaLISA immunoassay buffer, was added per well. The plate was read after second incubation for 45 min at RT in the dark. In pilot experiments, several gliadin-specific hmAbs were tested and gave similar results. The hmAbs UCD1114 1F03 and UCD1143 3B02 were chosen for the final experiments as they were among the commonly used VH/VL pairs VH3-23/VL4-69 and VH3-15/VK4-1, respectively.

### AlphaLISA screening of hmAbs for gliadin specificity

Anti-IgG AlphaLISA donor bead solution (1:400) and gliadin-specific hmAbs 1 μg ml^−1^ in AlphaLISA immunoassay buffer were incubated in 1.5 ml tube for 1 h at RT in the dark before 15 μl was transferred to each well in 384 well plate. Then 5 μl analyte was added per well, and the plate subsequently incubated for 30 min at RT in the dark. The analyte consisted of fixed concentration of 40 nM biotin-GSGSGS-PLQPEQPFP and titrations of either CT-gliadin, lysate of EBV-transfected B cells or a mixture of LPS (Sigma L-4391), CpG, recombinant TG2 (Phadia) and recombinant Jo-1 antigen (Phadia). After 45 min incubation at RT in the dark, AlphaScreen streptavidin donor bead solution was diluted 1:200 in AlphaLISA Immunoassay buffer and 15 μl added per well. The plate was read after 30 min incubation at RT in the dark.

## Additional information

**How to cite this article**: Steinsbø, Ø. *et al*. Restricted VH/VL usage and limited mutations in gluten-specific IgA of coeliac disease lesion plasma cells. *Nat. Commun.* 5:4041 doi: 10.1038/ncomms5041 (2014).

## Supplementary Material

Supplementary Information Supplementary Figures 1-2 and Supplementary Table 1
