# Supplementary material for: Restricted VH/VL usage and limited mutations in gluten-specific IgA of coeliac disease lesion plasma cells
Source: Nat Commun. 2014 Jun 9;5:4041. doi: 10.1038/ncomms5041 (PMC4059925; doi:10.1038/ncomms5041)
Supplement: Supplementary Information — Supplementary Figures 1-2 and Supplementary Table 1 [file ncomms5041-s1.pdf]

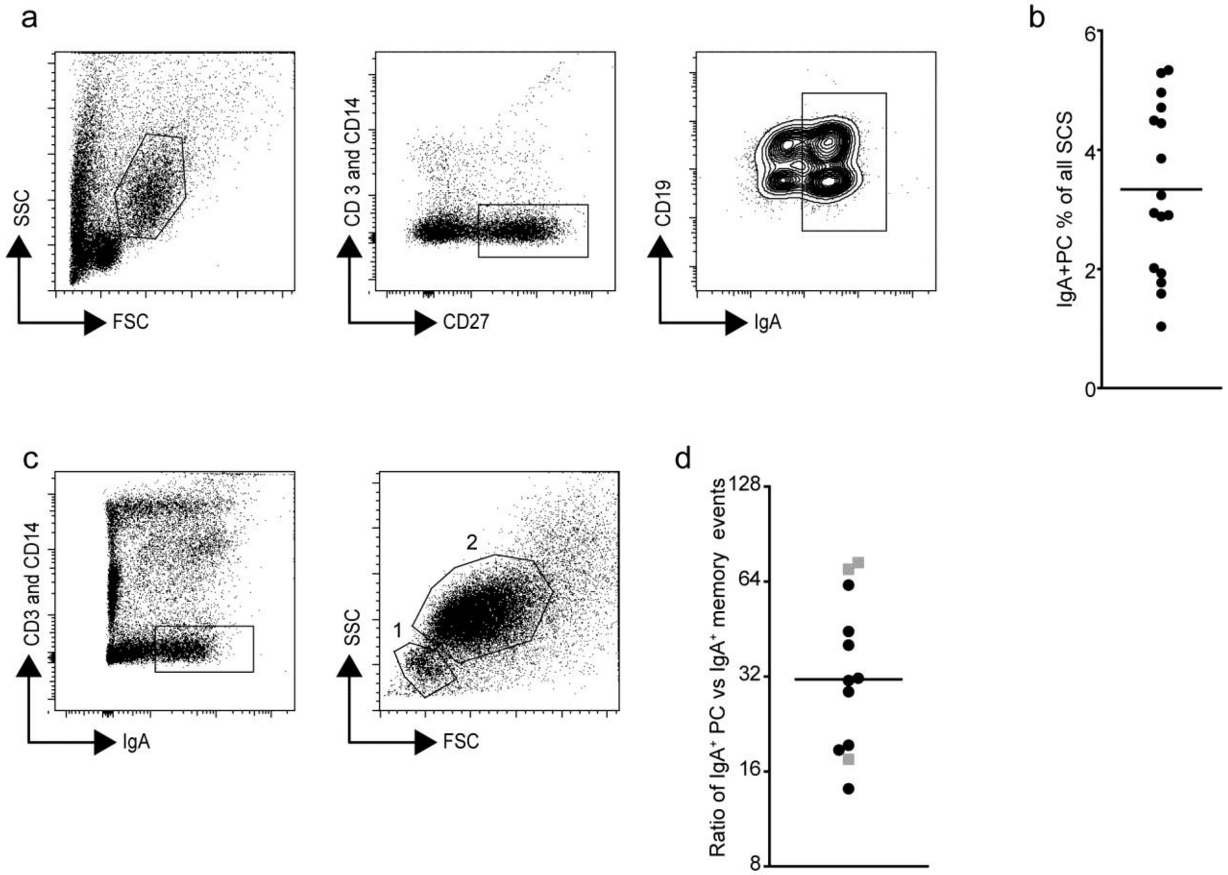

Supplementary Figure 1: Frequency of IgA^{+} PCs in SCSs of small intestinal biopsies. a) Representative flow plot of SCSs showing large, viable, CD3^{-}CD14^{-}CD27^{+}IgA^{+}, defined IgA^{+} PCs. b) Relative frequency of IgA^{+} PC of all cells in SCS in flow cytometry. Each dot represents one subject. Horizontal bar indicates mean value. c) Representative flow plot of SCSs of small intestinal biopsies. IgA^{+}CD3^{-}CD14^{-} gated events (left) analyzed in SSC/FSC plot to evaluate distribution of small events with memory B cell morphologies (1) versus large events with PC morphologies (2). d) Relative ratio of IgA^{+} PCs to IgA^{+} memory B cells. Each dot represents one subject. Grey squares are non-CD controls and black dots are UCD patients. Median value, indicated by horizontal bar is 31.5, with range 14-74, n = 12.

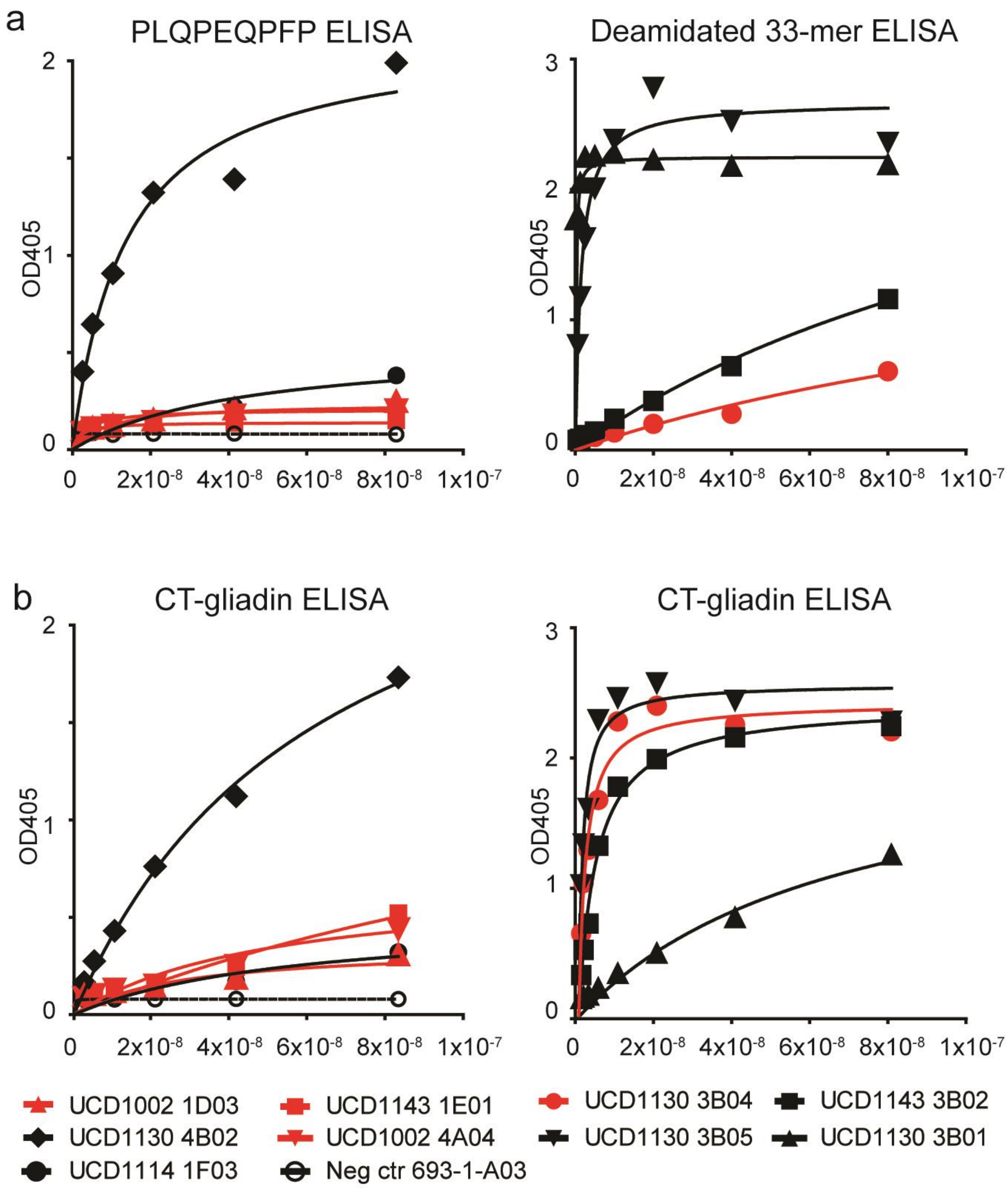

Supplementary Figure 2: Reactivity to antigen as depicted in headline measured by ELISA of 12 hmAbs cloned from single PLQPEQPFP^{+} or 33-mer^{+} IgA^{+} PCs. The hmAbs with reactivity in ELISA but no reactivity in AlphaLISA are represented by red curves. The concentrations of hmAbs are given on X-axis.

Patient number	Sorting antigen	hmAbs name	PLQPEQPFP reactivity in ELISA and/or AlphaLISA	DA 33-mer reactivity in ELISA and/or AlphaLISA	CT-gliadin reactivity in ELISA	Binding DA peptide >> NA peptide	VH gene	JH	CDR3 length	VH mutation	VL	JL/JK	CDR3 length	VL/VK mutation
UCD1002	PLQPEQPFP	1A05	YES	NO		NO	VH3-23	JH6	18	6	VL4-69	JL3	8	7
UCD1002	PLQPEQPFP	1B06	YES	YES		YES	VH3-15	JH4	14	16	VK4-1	JK4	9	1
UCD1002	PLQPEQPFP	1C02	YES	NO		NO	VH3-23	JH4	13	4	VL4-69	JL2	8	4
UCD1002	PLQPEQPFP	1D03	YES	NO		Not tested	VH3-74	JH4	14	4	VK4-1	JK1	9	0
UCD1002	PLQPEQPFP	1E01	YES	YES		NO	VH3-23	JH4	15	9	VL4-69	JL3	8	12
UCD1002	PLQPEQPFP	1E03	YES	NO		YES	VH3-15	JH4	14	7	VK4-1	JK4	10	2
UCD1002	PLQPEQPFP	1E05	YES	NO		NO	VH3-23	JH4	10	3	VL4-69	JL3	8	6
UCD1079	PLQPEQPFP	1A05	YES	NO		YES	VH3-15	JH5	13	9	VK4-1	JK1	10	7
UCD1079	PLQPEQPFP	1B02	YES	NO		NO	VH3-23	JH3	14	12	VL4-69	JL2	9	3
UCD1114	PLQPEQPFP	1F03	YES	YES		NO	VH3-23	JH4	11	5	VL4-69	JL1	9	5
UCD1114	PLQPEQPFP	1G01	YES	YES		YES	VH3-15	JH4	19	11	VK4-1	JK4	9	7
UCD1143	PLQPEQPFP	1B02	YES	YES		YES	VH3-21	JH6	19	6	VL2-14	JL2	8	7
UCD1143	PLQPEQPFP	1E01	YES	YES		NO	VH3-23	JH4	13	7	VL4-69	JL3	9	7
UCD1143	Deamidated 33-mer	3B02	YES	YES		YES	VH3-15	JH4	13	23	VK4-1	JK4	8	15
UCD1130	PLQPEQPFP	4A04	YES	YES		Not tested	VH3-73	JH4	10	9	VK4-1	JK4	8	2
UCD1130	PLQPEQPFP	4B02	YES	YES		NO	VH3-23	JH4	11	9	VL4-69	JL3	9	11
UCD1130	PLQPEQPFP	4B04	YES	NO		NO	VH3-23	JH3	12	20	VL4-69	JL3	9	9
UCD1130	PLQPEQPFP	4G05	YES	NO		YES	VH3-15	JH5	13	10	VK4-1	JK1	9	3
UCD1130	Deamidated 33-mer	3A02	YES	YES		NO	VH3-23	JH4	11	0	VL4-69	JL2	8	1
UCD1130	Deamidated 33-mer	3A04	YES	YES		NO	VH3-23	JH4	9	8	VL4-69	JL3	9	4
UCD1130	Deamidated 33-mer	3B01	YES	YES		2-3 Log	VH3-21	JH4	16	3	VK2-28	JK2	7	1
UCD1130	Deamidated 33-mer	3B04	YES	YES		NO	VH3-23	JH4	8	0	VL4-69	JL1	9	1
UCD1130	Deamidated 33-mer	3G05	NO	YES		YES	VH3-23	JH5	19	15	VK3-11	JK4	11	3
UCD1130	Deamidated 33-mer	3A05	YES	YES		Not tested	VH3-15	JH4	11	3	VK2-28	JK4	8	5
UCD1130	Deamidated 33-mer	3B03	NO	YES		YES	VH3-23	JH5	18	15	VK3-11	JK4	10	2
UCD1130	Deamidated 33-mer	3B05	YES	YES		1-2 Log	VH3-23	JH4	11	2	VK1-27	JK4	8	3

UCD1130	Deamidated 33-mer	3D01	YES	YES		Not tested	VH3-15	JH4	9	4	VK2-28	JK5	9	2
UCD1130	Deamidated 33-mer	3D05	YES	YES		1 Log	VH3-74	JH4	12	21	VK4-1	JK1	7	8
UCD1130	Deamidated 33-mer	3F01K	YES	YES		YES	VH3-15	JH4	7	17	VK4-1	JK3	8	5
UCD1130	Deamidated 33-mer	3G04	YES	YES		Not tested	VH3-21	JH4	16	7	VL2-14	JL2	8	14
UCD1130	CT-gliadin	2A02	YES	YES	YES	NO	VH3-23	JH3	16	6	VL4-69	JL2	9	8
UCD1065	CT-gliadin	4C01	NO	NO	YES	Not tested	VH3-15	JH4	10	13	VK3-20	JK2	9	5
UCD1065	CT-gliadin	4G05	NO	NO	YES	Not tested	VH3-15	JH6	12	12	VK3-20	JK1	8	12
UCD1050	CT-gliadin	5B05	YES	NO	YES	Not tested	VH3-72	JH4	10	4	VL2-11	JL1	11	3
UCD1186	CT-gliadin	1D01K	NO	YES	YES	YES	VH3-23	JH4	14	8	VK3-11	JK4	9	11
UCD1186	CT-gliadin	1C06L	YES	NO	YES	NO	VH3-23	JH4	15	12	VL4-69	JL3	9	11
UCD1030	CT-gliadin	1C02	YES	YES	YES	Not tested	VH4-4	JH4	15	12	VK4-1	JK1	9	8
UCD1163	CT-gliadin	1E04	YES	YES	YES	NO	VH3-23	JH3	20	3	VL4-69	JL2	9	3

Supplementary Table 1: Overview of the monoclonal antibodies from human gliadin-specific IgA. Donor subject (patient number) and isolation method (sorted by flow cytometry with selecting antigen PLQPEQPFP or deamidated 33-mer or isolated after in vitro culture with CT-gliadin as selecting antigen) are indicated as well as are reactivity to antigens, VH and VL usage and number of mutations.
